# A signature combining brain functional connectivity with executive and motor function for general cognitive decline in Parkinson’s disease

**DOI:** 10.3389/fneur.2025.1434733

**Published:** 2025-02-19

**Authors:** Jin Wang, Zhilin Shu, Yue Wang, Jiewei Lu, Xinyuan Zhang, Yuanyuan Cheng, Yang Yu, Jianda Han, Zhizhong Zhu, Ningbo Yu, Jialing Wu

**Affiliations:** ^1^Clinical College of Neurology, Neurosurgery and Neurorehabilitation, Tianjin Medical University, Tianjin, China; ^2^Department of Neurology, Tianjin Huanhu Hospital, Tianjin, China; ^3^Tianjin Key Laboratory of Cerebral Vascular and Neurodegenerative Diseases, Tianjin Neurosurgical Institute, Tianjin, China; ^4^College of Artificial Intelligence, Nankai University, Tianjin, China; ^5^Department of Rehabilitation Medicine, Tianjin Huanhu Hospital, Tianjin, China; ^6^Institute of Intelligence Technology and Robotic Systems, Shenzhen Research Institute of Nankai University, Shenzhen, China

**Keywords:** functional connectivity, functional near-infrared spectroscopy, cognitive decline, Parkinson’s disease, Stroop color-word test

## Abstract

**Introduction:**

Cognitive decline is common in Parkinson’s disease (PD). Reliance on neuropsychological testing alone can lead to delayed identification, and an objective and comprehensive approach is needed in clinical practice. We assessed brain functional connectivity during PD-MCI (mild cognitive impairment) and PD-NC (normal cognition) patients, and healthy controls (HC) completing the Stroop color-word test (SCWT) using functional near-infrared spectroscopy (fNIRS), and explored the predictive value of combining relevant brain function and behavioral information for general cognitive decline in PD.

**Methods:**

Nineteen patients with PD-MCI, 21 with PD-NC and 33 age-matched HC were recruited. Group differences in executive performance and prefrontal functional connectivity were analyzed. Receiver operating characteristic analysis was used to measure the value of combining brain functional connectivity with executive and motor function in predicting PD-MCI.

**Results:**

During the color-word incongruent test, PD-MCI patients had significantly lower correct rate than HC and PD-NC patients. Meanwhile, PD-MCI patients exhibited significantly increased regional strength of the left and right prefrontal cortex (RS_l_, RS_r_), and global efficiency than HC, and compared with PD-NC, PD-MCI patients showed significantly higher RS_r_. For PD patients, MMSE score and correct rate during the color-word incongruent test were negatively associated with the RS_r_ after adjusting for education level and age. After combined the RS_r_, correct rate and MDS-UPDRS III score, diagnostic sensitivity and specificity of PD-MCI reached 0.737 and 0.810, respectively, with an area under the curve of 0.830.

**Conclusion:**

We proposed a signature combining brain functional connectivity with executive and motor function for general cognitive decline in PD, which could provide new insights into early detection and intervention of this problem.

## 1 Introduction

Cognitive decline including mild cognitive impairment (MCI) and dementia is frequently found in patients with Parkinson’s disease (PD). PD-MCI is an independent risk factor for dementia and is considered a critical stage of rehabilitation treatment (1). PD-MCI involves multiple domains like executive function, visuospatial and memory abilities and is characterized by executive deficits, including impaired conflict detection, selective attention, and inhibitory functions, which may have significant adverse effects on patients’ social functioning and quality of life (2). In addition, there is a strong correlation between impaired executive function and decreased motor capacity in PD. (3) For example, Nie et al. (4) collected 234 non-dementia PD patients for analysis of relevant risk factors of PD-MCI, and found that increased UPDRS-III score (OR: 1.032; 95%CI: 1.008–1.057; *p* = 0.01) was a risk factor for PD-MCI. A meta-analysis by Baiano et al. (1) demonstrated that PD-MCI patients had worse motor symptoms than non-PD-MCI patients, and the ES was significantly positive (=0.40). However, PD-MCI is often overlooked by patients, their families, and even clinicians due to patients’ retained functional independence and more prominent motor symptoms. Additionally, the potentially lagging changes in neuropsychological tests, when compared with those in brain function, may also impede the early identification of the disease (5). Hence, developing an improved and objective method combining relevant brain function and behavioral features to evaluate PD-MCI is of great importance.

Functional connectivity (FC) analysis can reflect the communication between different brain regions. A few resting-state functional MRI (rs-fMRI) researches have suggested that patients with PD-MCI demonstrate increased FC between right inferior frontal gyrus and posterior cingulate cortex (6, 7), and cognitive training can help PD patients save brain resources (8). Moreover, De Micco et al. (9) showed that FC within frontal, parietal, temporal, sensorimotor and occipital networks using rs-fMRI could independently predict cognitive progression at 2-year follow-up.

However, there is limited information regarding cortical FC of specific areas during cognitive processes in PD. In the past few years, functional near-infrared spectroscopy (fNIRS) has been increasingly applied in cortical activation and neural networks researches with its unique advantages of task suitability, and our team’s previous study has shown its effectiveness on reflecting cognitive impairment (10). But at that time, the cortical abnormalities caused by PD itself were not fully considered, so the PD-NC (normal cognition) group was not included, and the inclusion of brain channels was not targeted enough. Therefore, we could use fNIRS to study FC in the prefrontal cortex (PFC) networks, a core brain region responsible for executive function, during the Stroop color-word test (SCWT) in PD-MCI and PD-NC patients.

Consequently, we aimed to study the characteristics of brain FC during cognitive tasks in PD patients and explore the diagnostic value of its combination with other necessary features for PD-MCI.

## 2 Materials and methods

### 2.1 Participants

Our study enrolled 40 patients with PD from the out- and inpatient departments of Tianjin Huanhu Hospital and 33 healthy controls (HC) were recruited from among the patients’ caregivers. All patients were clinically diagnosed with PD according to the criteria of MDS (2015) (11) and did not meet Parkinson’s disease dementia (12). The criteria for inclusion for PD patients were: aged ≥50 years; at Hoehn and Yahr (H&Y) stages I to III in their “off” state; received stable medication for more than 2 months; able to understand and coordinate with the entire examination and testing process; and being right-handed. Exclusion criteria comprised: being illiterate; having color vision disorders; suffering from Parkinson’s disease psychosis, like hallucinations; obvious depression (Beck Depression Inventory (BDI) score > 13); recent use of anticholinergic or other drugs affecting cognition; serious motor complications; prior deep brain stimulation surgeries; and histories of stroke, hypothyroidism, hydrocephalus or other diseases that may affect cortical blood flow and cognition. All participants in the HC group were also ≥50 years old, right-handed, not illiterate, free from color vision disorders, not in an obvious depressive state, had no histories of diseases affecting cortical blood flow, and demonstrated good compliance. Moreover, neither the participants themselves nor their families reported any cognitive decline in the HC group.

The enrolled PD patients were divided into PD-MCI group and PD-NC group. PD-MCI was determined using the MDS Task Force Level I criteria (13): (1) gradual decline in cognitive function based on PD as reported by patients or observers; (2) MoCA (Beijing Version) confirmed evidence of objective cognitive impairment. Based on a large-scale epidemiological survey in China, the cutoff point was set at 19/20 for individuals whose schooling duration was ≤6 years and 24/25 for those with ≥7 years of education (14); (3) and although they may have some difficulty in processing complex functional tasks, cognitive dysfunction was not sufficient to significantly affect their functional independence. Patients who did not meet PD-MCI were assigned to the PD-NC group. Correspondingly, the healthy subjects we finally included also did not meet the MCI criteria. Specifically, the MoCA threshold followed the same rules as those for PD-MCI.

The sample size was computed using G*Power v3.1.9.2. An estimated sample size of 66 subjects was deemed sufficient. However, considering the potential for missing data, a total of 73 subjects were enrolled in this study, which means an increase of approximately 10%.

The Ethics Committee of Tianjin Huanhu Hospital approved this study, which was then registered in the Chinese Clinical Trial Registry. Before participating in the research, all subjects gave their written informed consent.

### 2.2 Experimental procedures

**Figure 1:**
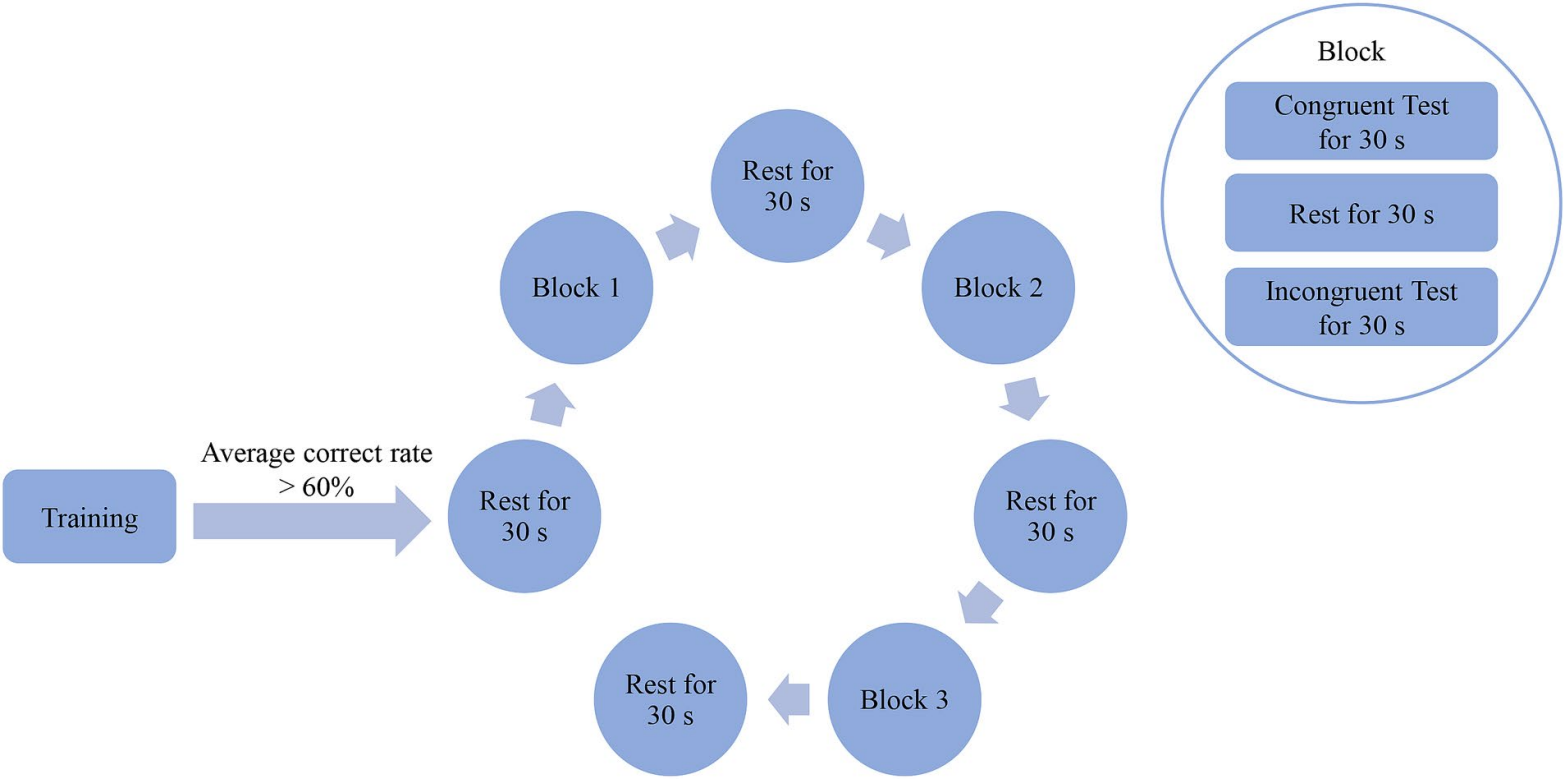
Entire experimental procedure of the SCWT. Subjects were required to read the colors (including red, yellow, green and blue) in which words (red, yellow, green or blue) on the middle of the screen were written rather than words themselves. Color-word congruent or incongruent test denoted that colors of words exactly coincided with or differed from meanings of words themselves. Each subject was required to have an average correct rate of >60% by training. And after a rest for 30 s, three blocks were conducted with each followed by a 30-s of rest. Each block contained one color-word congruent test, one color-word incongruent test and a rest between them with each lasting 30 s. Color-word congruent and incongruent tests were generated randomly.

The experimental procedure is illustrated in Figure 1. All PD patients (in the “off” state) and HC independently completed the computerized SCWT. For all subjects, the same experimenter controlled the computer. The test required them to read the colors (including red, yellow, green and blue) in which the words “red,” “yellow,” “green,” or “blue” in the middle of the screen were written, rather than the words themselves. Once a word was read out, the next one was showed immediately. Color-word congruent or incongruent test denoted that color of words exactly coincided with or differed from the meanings of the words. Each subject was required to have an average correct rate of >60% by training. And after a rest for 30 s, three blocks were conducted, with each followed by a 30-s of rest. Each block contained one color-word congruent test, one color-word incongruent test and a rest between them with each lasting 30 s. Color-word congruent and incongruent tests were generated randomly. In addition, the background of the screen was always black, and the experiment was conducted in a controlled laboratory setting to minimize potential environmental effects. Moreover, the amounts of total responses and correct responses, and correct rate during the color-word congruent and incongruent tests were calculated, respectively.

### 2.3 fNIRS data acquisition and preprocessing

**Figure 2:**
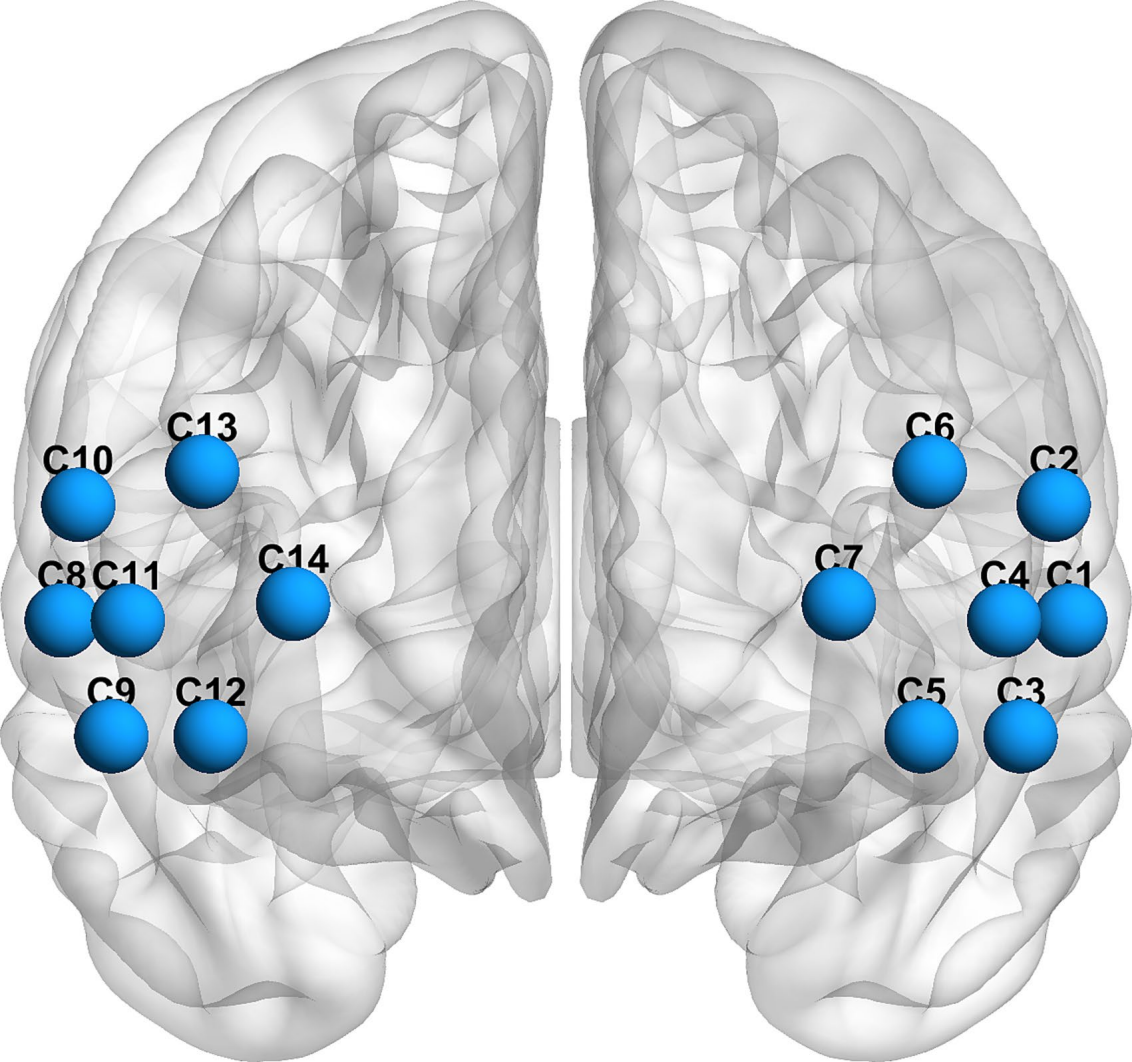
Deployments of the fNIRS channels in LPFC and RPFC.

fNIRS scanning was performed using a wireless continuous-wave system (Nirsmart, produced by Danyang Huichuang Medical Equipment Co., Ltd.) when participants performing the SCWT. The system made use of near-infrared light at 730 and 850 nm to gauge the optical intensities of oxygenated hemoglobin (HbO2) and deoxygenated hemoglobin (HbR) at a rate of 11 samples per second. 6 sources, 6 detectors and the resulting 14 channels were placed on the left and right PFC (LPFC, RPFC). The source-detector distance was 3 cm. The instrument was designed in accordance with the three-dimensional positioning algorithm and international 10–20 electrode placement system. This is illustrated in Figure 2. The identification of interest regions was grounded on the standardized cap sizes to accommodate various cephalic circumferences.

For the raw fNIRS data, two methods were used to remove the motion artifacts: moving standard deviation and spline interpolation. Subsequently, a bandpass filter between 0.01–0.2 Hz was used to suppress physiological noise such as pulse, respiration, and baseline drift. Then, HbO2 concentration variations were computed using the modified Beer–Lambert law. Finally, the HbO2 data for the task periods were extracted for further analysis.

### 2.4 FC measures

The HbO2 data of each kind of SCWT, congruent or incongruent, was divided into three 30-s epochs of the task periods and averaged to form a new 30-s epoch. Pearson’s correlation coefficient (PCC) between each pair of channels was calculated for the averaged epoch to reflect the functional correlation of the brain regions. Given two HbO2 data, u and v , the PCC values ( $P_{uv}$ ) was calculated as follows:


$$P_{uv}=\frac{cov\left(u,v\right)}{\sigma_{u}\sigma_{v}}$$

Where, $cov\left(u,v\right)$ represents the covariance between u and $v;\mathrm{and}\;\sigma_{u}$ and $\sigma_{v}$ denote the standard deviations of u and v respectively.

Fisher’s Z-transformation was applied to transform $P_{uv}$ values to $Z_{uv}$ values, namely Z -transformed PCC values between u and v , which normalized the distribution and decreased the skewness.


$$Z_{uv}=\frac{1}{2}\ln\left(\frac{1+P_{uv}}{1-P_{uv}}\right)$$

Considering that the meaning of negative connections was not yet well-defined, and given that our focus was on the integration of brain information during cognitive tasks, all negative connections and self - connections were set to zero.

A network was constructed when channels were regarded as nodes and the PCC values between every two channels were treated as edges. In this study, three indices were introduced to quantify the characteristics of brain networks: regional strength ( RS ), global efficiency ( GE ) and clustering coefficient ( CC ).

$RS_{rg}$ is considered to reflect the strength of connections between a brain region and other brain regions and within the region itself (15). It was worked out as follows:


$$RS_{rg}=\frac{1}{N_{rg}}\underset{i\in G_{rg}}{\sum }k_{i}$$

Where, $k_{i}$ refers to the degree of node i , $G_{rg}$ and $N_{rg}$ indicate the set of all nodes belonging to a certain region and the number of nodes, respectively.

GE is a measure of the functional integration of brain networks (16). It was calculated as follows:


$$GE=\frac{1}{N}\underset{i\in N}{\sum }\frac{\sum_{j\in G,j\neq i}{\left(d_{ij}\right)}^{-1}}{N-1}$$

Where, $d_{ij}$ stands for the inverse of the average shortest path length between each of the two channels ( i and j ), G and N are the collections of all nodes and the total node count, respectively.

CC was introduced to depict the local efficiency of information transfer (17). It was defined as follows:


$$CC=\frac{1}{N}\overset{N}{\underset{i=1}{\sum }}\frac{2t_{i}}{k_{i}\left(k_{i}-1\right)}$$

$$t_{i}=\frac{1}{2}\underset{\begin{array}{c} j,k\in G,\\ j\neq k\neq i \end{array}}{\sum }z_{ij}z_{jk}z_{ki}$$

Where, $t_{i}$ signifies the number of triangles that can be formed between node i and its neighboring nodes.

The FC was analyzed using MATLAB (2018b, MathWorks, Natick, Massachusetts) and the measures was derived using Brain Connectivity Toolbox.

### 2.5 Statistical analysis

The Shapiro–Wilk test was used to check whether the data within each group followed a normal distribution, if necessary. Normally distributed data, were expressed by means (standard deviations); if not, by medians (lower quartiles-upper quartiles). The *t*-test, one-way analysis of variance (ANOVA), chi-square, Mann–Whitney *U*, Kruskal-Wallis and Wilcoxon signed-rank tests were used to assess between-group differences in the demographic, clinical, neuropsychological, and FC data. Bonferroni’s adjustment was used for multiple comparisons between the groups. Multiple linear regression method was used to examine the relationship between FC and MMSE scores, MoCA scores as well as executive performance during the SCWT. PD-MCI was taken as the gold standard to draw the receiver operating characteristic (ROC) curve. The area under the curve (AUC) was calculated to evaluate the quality of each predictor. *p* ≤ 0.05 was considered statistically significant. The data were analyzed using SPSS version 25 for Windows.

## 3 Results

### 3.1 Demographic, neuropsychological and clinical characteristics

In this study, a total of 73 participants were enrolled, including 33 HC, 21 patients with PD-NC and 19 with PD-MCI. The demographic, neuropsychological and clinical data are presented in Table 1. There were 12 patients with PD at H&Y stage II and 28 at stage III, with an average MDS-UPDRS III score at 35.53 (13.17). No significant differences regarding age or sex were identified among the three groups. In terms of education, the years of schooling in PD-NC group were significantly higher than those of the HC group (*p* = 0.016) and PD-MCI group (*p* = 0.019). Patients with PD-MCI had significantly lower MMSE (*p* = 0.004, *p* < 0.001, respectively) and MoCA scores (*p* < 0.001, respectively) than HC and PD-NC patients. Patients with PD-MCI exhibited significantly higher proportion of H&Y stage III and higher MDS-UPDRS III scores than PD-NC patients (*p* = 0.011, *p* = 0.010, respectively). There were no statistically significant differences in disease duration, side of more affected limb, or levodopa equivalent daily dosage (LEDD) between the PD-NC and PD-MCI groups.

**Table 1:** Comparisons of demographic, neuropsychological and clinical characteristics among HC, PD-NC and PD-MCI groups^a^.

Characteristics	HC (*n* = 33)	PD-NC (*n* = 21)	PD-MCI (*n* = 19)	*p*-value
Age (years)	61.42 (6.41)	62.24 (8.83)	64.84 (8.57)	0.308
Sex (male/female)	13/20	11/10	13/6	0.133
Education (years)	9 (7–9)	12 (9–12.5)^#^	7 (7–12)	**0.007**
MMSE (score)	28 (27–29)	28 (28–30)	26.32 (1.95)^*^	**<0.001**
MoCA (score)	24.76 (2.33)	25 (25–26.5)	22 (18–24)^*^	**<0.001**
Disease duration (years)^b^	–	3.98 (1.95)	4.76 (1.40)	0.155
More affected limb (left/right)	–	8/13	8/11	0.796
H&Y stage (II/III)^c^	–	10/11	2/17	**0.011**
MDS-UPDRS III (score)^c^	–	30.52 (12.35)	41.05 (12.04)	**0.010**
LEDD (mg/d)	–	375 (325–425)	425 (375–600)	0.089

H&Y stage, Hoehn and Yahr stage; LEDD, levodopa equivalent daily dosage.

a Data are presented as mean (SD) or median (lower quartile-upper quartile).

b Data are calculated since date of the initial motor symptom.

c Data are obtained during patients’ “off” state.

# Indicates significant difference from the HC group and PD-MCI group.

* Indicates significant difference from the HC group and PD-NC group.

Values with *p* ≤ 0.05 are bolded for indication.

### 3.2 Cognitive performance during the SCWT

A detailed summary of cognitive performance during the SCWT for HC and patients with PD-NC and PD-MCI is provided in Table 2. As expected, amounts of total responses and correct responses, and correct rate in the color-word congruent test were significantly higher than those in the incongruent condition for the HC (*p* < 0.001, *p* < 0.001, *p* = 0.001), PD-NC (*p* < 0.001, respectively) and PD-MCI groups (*p* < 0.001, respectively), indicating the Stroop effect. None of the measures of cognitive performance in the congruent condition were statistically significant among the three groups. However, during the color-word incongruent test, patients with PD-MCI exhibited significantly worse performance with reduced correct rate, compared to HC (*p* = 0.001) and patients with PD-NC (*p* = 0.046).

**Table 2:** Comparison of cognitive performance during the SCWT among HC, PD-NC and PD-MCI groups^a^.

Characteristics	HC (*n* = 33)	PD-NC (*n* = 21)	PD-MCI (*n* = 19)	*p*-value
C-total responses	59.00 (53.50–79.50)	59.48 (11.48)	60.79 (13.50)	0.603
C-correct responses	62.70 (15.35)	59.38 (11.53)	60.74 (13.48)	0.683
C-correct rate	1.00 (1.00–1.00)	1.00 (1.00–1.00)	1.00 (1.00–1.00)	0.849
Inc-total responses	50.76 (9.94)	48.33 (7.91)	46.68 (12.25)	0.356
Inc-correct responses	49.06 (10.37)	46.33 (8.63)	43.37 (12.85)	0.180
Inc-correct rate	0.98 (0.94–1.00)	0.96 (0.95–0.98)	0.92 (0.04)^*^^,^^#^	**0.001**

C-, color-word congruent test-; Inc-, color-word incongruent test-.

a Data are presented as mean (SD) or median (lower quartile-upper quartile).

* Indicates significant difference from the HC group.

# Indicates significant difference from the PD-NC group.

Values with *p* ≤ 0.05 are bolded for indication.

### 3.3 FC measures during the SCWT

**Figure 3:**
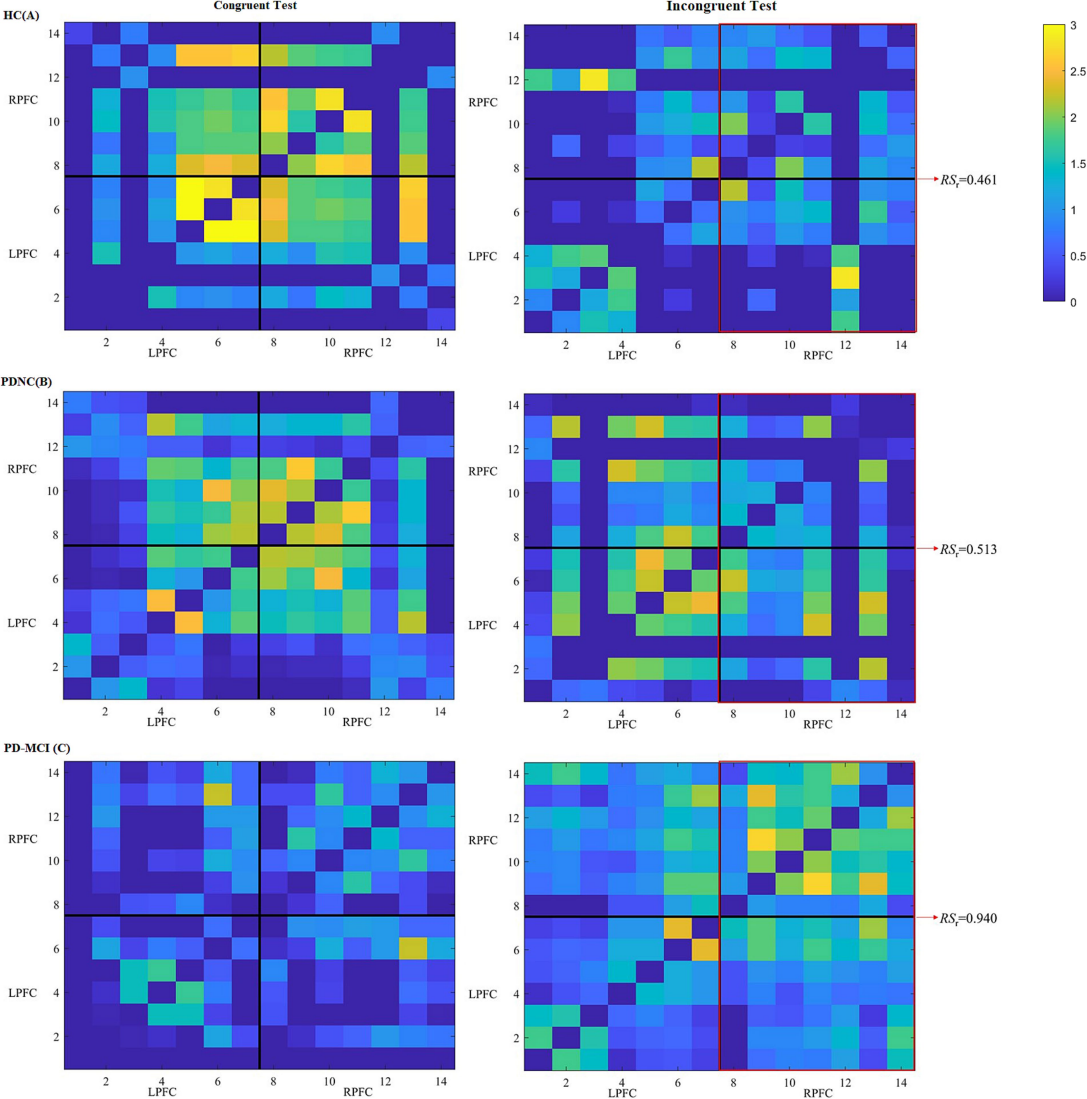
Heatmaps of FC among different channels of three participants, respectively, from HC **(A)**, PD-NC **(B)** and PD-MCI **(C)** groups during the color-word congruent (left) and incongruent (right) tests. The heatmaps are divided into LPFC and RPFC parts, and *x*-axis and *y*-axis correspond to the fNIRS channels. Each pixel denotes the Z -transformed Pearson’s correlation coefficient value between the paired channels. Warmer colors denote larger values between the brain regions, whereas colder colors denote smaller values. The red frames reflect connections between channels in the RPFC and LPFC and connections within the RPFC.

In terms of FC measures, compared to completing the color-word incongruent test, the HC group showed a consistently higher tendency of RS of the LPFC and RPFC (RS_l_, RS_r_), as well as GE and CC during the color-word congruent test, whereas the PD-MCI group displayed the opposite trend. However, no significant differences were found between the color-word congruent and incongruent tests for all the three groups. During the color-word congruent test, no significant differences were found among the three groups. Nevertheless, in the color-word incongruent test, compared to HC and PD-NC, PD-MCI showed an increasing trend of all the FC measures and the differences in RS_l_ (*p* = 0.030), RS_r_ (*p* = 0.009), and GE (*p* = 0.007) reached statistical significance between the PD-MCI and HC groups, and RS_r_ was the only parameter that differed significantly between the PD-MCI and PD-NC patients (*p* = 0.044). These results are shown in Tables 3, 4 and Figure 3.

**Table 3:** Comparison of functional connectivity during the SCWT among HC, PD-NC and PD-MCI groups^a^.

Characteristics	HC (*n* = 33)	PD-NC (*n* = 21)	PD-MCI (*n* = 19)	*p*-value
C-RS_l_	0.81 (0.34)	0.81 (0.38)	0.87 (0.39)	0.822
C-RS_r_	0.85 (0.36)	0.78 (0.57–0.95)	0.88 (0.34)	0.826
C-GE	0.96 (0.33)	0.84 (0.70–1.15)	1.00 (0.36)	0.827
C-CC	0.38 (0.09)	0.37 (0.08)	0.38 (0.09)	0.856
Inc-RS_l_	0.69 (0.26)	0.73 (0.23)	0.94 (0.35)^*^	**0.035**
Inc-RS_r_	0.72 (0.29)	0.75 (0.26)	0.98 (0.33)^*^^,^^#^	**0.008**
Inc-GE	0.83 (0.26)	0.88 (0.23)	1.08 (0.33)^*^	**0.008**
Inc-CC	0.35 (0.09)	0.34 (0.07)	0.39 (0.10)	0.141

C-, color-word congruent test-; Inc-, color-word incongruent test-; RS_l_, regional strength of the left prefrontal cortex; RS_r_, regional strength of the right prefrontal cortex; GE, global efficiency; CC, clustering coefficient.

a Data are presented as mean (SD) or median (lower quartile-upper quartile).

* Indicates significant difference from the HC group.

# Indicates significant difference from the PD-NC group.

Values with *p* ≤ 0.05 are bolded for indication.

**Table 4:** Comparison of brain functional connectivity between the color-word congruent test and color-word incongruent test (*p*-value) in three groups.

Groups	*p-RS_l_*	*p-RS_r_*	*p-GE*	*p-CC*
HC	0.075	0.051	0.054	0.150
PD-NC	0.289	0.274	0.322	0.198
PD-MCI	0.441	0.226	0.310	0.588

RS_l_, regional strength of the left prefrontal cortex; RS_r_, regional strength of the right prefrontal cortex; GE, global efficiency; CC, clustering coefficient.

### 3.4 Correlation between cognitive function and RS_r_ in PD patients

In the uncorrected model, MMSE score, MoCA score, and correct rate during the color-word incongruent test were all negatively correlated with the RS_r_. Considering that the PD-NC group had significantly different years of education from the other two groups, we adjusted for this and the age. After adjusting, we found that MMSE score and correct rate were still associated with the RS_r_. These results are shown in Table 5.

**Table 5:** Correlation between cognitive function and RS_r_ level (color-word incongruent test) in PD patients.

Characteristics	Model 1				Model 2			
	*β*	95% CI	SE	*p*-value	*β*	95% CI	SE	*p*-value
MMSE	−0.075	−0.122 ~ −0.028	0.023	0.003	−0.101	−0.159 ~ −0.044	0.028	0.001
MoCA	−0.034	−0.069 ~ 0.000	0.017	0.050	−0.042	−0.085 ~ 0.001	0.021	0.056
Inc-correct rate	−2.244	−4.449 ~ −0.038	1.089	0.046	−2.331	−4.593 ~ −0.068	1.116	0.044

Inc-, color-word incongruent test-. Model 1: unadjusted model. Model 2: adjusted for education and age.

### 3.5 ROC analysis

**Figure 4:**
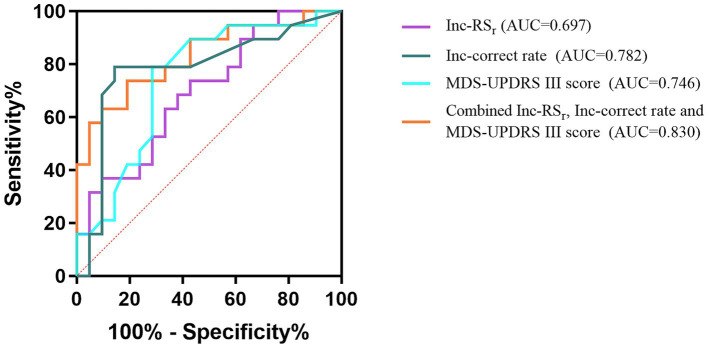
ROC curves of the Inc-RS_r_, Inc-correct rate, MDS-UPDRS III score, and the combined parameter for PD-MCI. Inc-, color-word incongruent test-.

ROC analysis was performed for RS_r_, correct rate during the color-word incongruent test and score of MDS-UPDRS III. The AUC of these parameters for detecting PD-MCI from non-dementia patients with PD were 0.697, 0.782, and 0.746, respectively. The above three parameters were jointly analyzed to assess their diagnostic value. The AUC of the combined parameter for detecting PD-MCI was 0.830, with cutoff value of 0.548, sensitivity of 0.737, and specificity of 0.810. These results are shown in Figure 4.

## 4 Discussion

### 4.1 Executive and motor deficits in PD-MCI patients

Our study confirmed a significant decline in executive function in patients with PD-MCI compared to HC and PD-NC patients. This could be mainly caused by the related pathogenesis like formation of cortical Lewy bodies, Alzheimer’s disease-like changes, cerebral microvascular lesions, and various neurotransmitter changes dominated by dopamine. Previous studies have found that older age, males, lower education level, longer disease course, and severe motor symptoms are associated with the occurrence of PD-MCI (18). In our study, there were no significant differences in age, gender or disease course between the PD-MCI and PD-NC groups, but patients with PD-MCI had significantly higher MDS-UPDRS III scores compared with PD-NC patients, with 41.05 (12.04) and 30.52 (12.35) points, respectively. Hence, in combination with previous researches conducted by Nie (4), Baiano et al. (1), MDS-UPDRS III score could be used as an effective supplementary indicator to reflect the cognitive status of PD patients.

According to the Chinese population-based study (14) which focused on the stratification of educational level, the average MoCA scores of our HC, PD-NC and PD-MCI groups are reasonable, although they are much lower than those of the original research. Xu’s study (19) and Jia’s (20) study can prove this. In China, MoCA with its good validity and reliability has become one of the most recommended methods for the general cognitive function assessment of Parkinson’s disease. However, it is obviously affected by the educational level, which is prone to causing deviation. Hence, when using MoCA to reflect cognitive function, we have fully considered this. In China, for the age range included in our study, low educational level is common, and the influence it brings is multifaceted. Firstly, older people with a low educational level are inclined to only handle simple family affairs in daily life and have a low degree of social participation. That is to say, the cognitive requirements for them are not high enough. And if it is a PD patient, this situation will be more prominent. Secondly, some items of the MoCA heavily depend on the educational level, especially the line-connecting, clock-drawing, cube-copying, repetition 1, language fluency, similarity 2, and delayed recall items (21). To sum up, the common low educational level, the resulting reduction in the complaint of cognitive decline which is crucial for distinguishing MCI and NC, and the unfriendliness of MoCA towards people with low educational level jointly leads to much lower MoCA scores than those in foreign countries, no matter it is HC, PD-NC or PD-MCI.

### 4.2 FC during the SCWT in PD patients

To understand PD-MCI from the perspective of brain function was one of our objectives. The FC analysis indicated that patients with PD-MCI had significant higher RS_l_, RS_r_, and GE than HC during the color-word incongruent test, and the RS_r_ was found to achieve a predictive value in differentiating PD-MCI from PD-NC. RS can be considered to directly reflect the intensity of information processing of a particular brain region, and GE is a superior measure of resource integration. We demonstrated that RS_r_, not RS_l_ was a promising indicator in PD-MCI. This may involve complex inhibition and attention networks. The neural basis of inhibitory function is a right-sided framework consisting of anatomically connected presupplementary motor area, inferior frontal gyrus and subthalamic nucleus (22, 23). Attentional function is mainly managed by the ventral and dorsal networks. The ventral network, consisting of right frontoparietal regions, like inferior frontal gyrus, is responsible for attention shifting, and the dorsal network, involving bilateral frontoparietal regions, is responsible for the maintenance of alertness via the top-down allocation of attention (24, 25). Therefore, the right neural basis of the SCWT can be explained to some extent. Additionally, there was no significant difference in CC among the three groups. This can be explained from the following two aspects: On the one hand, when participants perform a new complex task, brain synchronization pattern is formed first, resulting in higher integrated components rather than separate components, and the robustness of the brain network to random errors and the local efficiency of information processing cannot mature in a short time. That is, CC changes relatively little under our cognitive test. On the other hand, the cognitive decline of PD-MCI patients was mainly in fluid intelligence, which is mainly related to the integration of brain functional network (26). Further research on the role of tasks that repeatedly stimulate the frontal cognitive networks over longer periods of time on CC and other FC measures may aid in elucidating this issue.

In this study, MMSE score and correct rate during the color-word incongruent test were negatively associated with the RS_r_ after adjusting for education level and age, suggesting a compensatory mechanism. Klobušiaková et al. (27) evaluated the between-network connectivity of the frontoparietal control network and other resting state networks in patients with PD-MCI and PD-NC using fMRI, and found that the connectivity increased with time as well as MCI status, also indicating similar principle.

This study revealed that although not statistically significant, HC group displayed an accordant higher trend of RS_l_, RS_r_, GE and CC when performing the color-word congruent test compared to the incongruent condition, and PD-MCI group exhibited stronger FC during the color-word incongruent test behind the common better performance in the congruent condition for the two groups. This is possibly because HC had greater mental flexibility and information monitoring when completing the SCWT. It should be more difficult to distinguish the two dimensions of word color and word meaning when they are consistent. For similar information, they may need to pay more attention and overcome self-doubt. In contrast, for inconsistent information, they would pay more attention to the target while ignoring others and less consideration to the contradiction and their usual reaction tendency (28).

A study on executive function in PD patients using fMRI by Hamada et al. (29) showed that reduced switching in semantic fluency task was a strong indicator of PD-MCI and was correlated to decreased FC in the salience network. The different FC results might be related to several differences between our study and theirs. First, the range of brain regions involved in the salience network is larger in their study. Second, compared with the task state, their resting-state network may be more vulnerable to the influences of individual brain development trajectories and emotional regulation, which may have affected the results to some extent. Third, although both are focused on executive function, compared with the verbal fluency task, SCWT is likely to place more emphasis on reflecting the abilities of control processing and selective attention, and may be less affected by the educational level of the participants.

### 4.3 ROC analysis in predicting PD-MCI

Although FC, which reflects changes in central function, is generally considered to be more sensitive than behavioral manifestations, the RS_r_ was not an ideal classifier when used alone to predict PD-MCI in our study (AUC = 0.697). This may be due to the fact that it is not only influenced by cognitive function, but also related to individual effort levels, different thinking habits, and ceiling and floor effects. Therefore, the corresponding cognitive performance and indicators that better reflect the pathological nature of PD-MCI should be combined. Changes in the fronto-striatal pathway and dopaminergic and non-dopaminergic transmitters lead to impaired cognitive and motor function in PD. Relatively speaking, the decline in motor ability is more directly affected and easier to detect in actual clinical work. In addition, as mentioned above, the suggestive value of more severe motor deficits for PD-MCI has been demonstrated. Hence, we used RS_r_ and correct rate during the color-word incongruent test, and MDS-UPDRS III score for joint analysis. The AUC of the combined parameter for detecting PD-MCI reached 0.830, with cutoff value of 0.548, sensitivity of 0.737, and specificity of 0.810.

This study had some limitations. (1) It had a cross-sectional design with a small sample size, which may limit the generalizability of our findings. Longitudinal and larger studies are needed to confirm these results. (2) The PD-NC group in our study had significantly more years of education compared to the PD-MCI and HC groups. This disparity undermines the optimal comparability of the baseline information across the three groups. (3) When analyzing brain FC, we set all negative connections and self-connections to zero, which could lead to the loss of some inhibitory regulation information. Additionally, we did not adopt methods to eliminate the interference of scalp hemodynamics in the fNIRS data. In future research, we intend to handle these issues with more prudence and explore alternative or more refined methods. (4) Posterior parietal cortex also plays a role in encoding targets and goal-related information for control, and we can incorporate it into further research. (5) The SCWT we designed has a fixed duration, which may not be sensitive enough to reflect subjects’ reaction time by analyzing the amounts of responses. (6) When judging MoCA abnormalities, we chose to refer to a large-scale epidemiological study in China with similar wide range of educational levels, but our age was slightly younger, which might affect the division of PD-MCI and PD-NC.

## 5 Conclusion

In conclusion, we provide a novel signature that combines brain FC with executive and motor function to study cognitive decline in PD. Our findings may help promote early rehabilitation and follow up.

## Data Availability

The raw data supporting the conclusions of this article will be made available by the authors, without undue reservation.
